# The entire chloroplast genome sequence of *Asparagus setaceus* (Kunth) Jessop: Genome structure, gene composition, and phylogenetic analysis in Asparagaceae

**DOI:** 10.1515/biol-2022-0497

**Published:** 2022-11-23

**Authors:** Quan Kuang, Wentao Sheng

**Affiliations:** Department of Biological Technology, Nanchang Normal University, Nanchang, 330032, Jiangxi, China

**Keywords:** chloroplast genome, *Asparagus setaceus*, PacBio and Illumina sequencing, phylogenetic tree analysis

## Abstract

*Asparagus setaceus* (Kunth) Jessop is a horticultural plant of the genus *Asparagus*. Herein, the whole chloroplast (cp) genome of *A. setaceus* was sequenced with PacBio and Illumina sequencing systems. The cp genome shows a characteristic quadripartite structure with 158,076 bp. In total, 135 genes were annotated, containing 89 protein-coding, 38 tRNA, and 8 rRNA genes. Contrast with the previous cp genome of *A. setaceus* registered in NCBI, we identified 7 single-nucleotide polymorphisms and 15 indels, mostly situated in noncoding areas. Meanwhile, 36 repeat structures and 260 simple sequence repeats were marked out. A bias for A/T-ending codons was shown in this cp genome. Furthermore, we predicted 78 RNA-editing sites in 29 genes, which were all for C-to-U transitions. And it was also proven that positive selection was exerted on the *rpoC1* gene of *A. setaceus* with the *K*_a_/*K*_s_ data. Meanwhile, a conservative gene order and highly similar sequences of protein-coding genes were revealed within *Asparagus* species. Phylogenetic tree analysis indicated that *A. setaceus* was a sister to *Asparagus cochinchinensis*. Taken together, our released genome provided valuable information for the gene composition, genetics comparison, and the phylogeny studies of *A. setaceus*.

## 1 Introduction

Chloroplast (cp) is the organ of photosynthesis in plant cells. The cp genome plays a key role in plant evolution, growth, and development [1]. In general, its genome is a circular double-stranded DNA molecule with a length of several hundred kilobases (kb). In its structure composition, the cp genome is mainly made up of four independent regions, namely consisting of a large single copy (LSC) region, two separate inverted repeat (IRa/IRb) regions, and a small single copy (SSC) region [2]. Based on the characteristics of its small genome size, conserved genome structure, and gene composition, the cp genomic sequences have supplied abundant data that are helpful for resolving the phylogenetic relationship in plant taxonomy [3].

*Asparagus setaceus* (common name: Asparagus fern) is a useful ornamental plant affiliated with the genus *Asparagus* in Asparagaceae. This genus includes both hermaphrodite and dioecious plants, which can be considered as an ideal genus for the sex chromosome origin and its phylogenetic relationship studies [4]. As a representative plant in this genus, *A. setaceus* is a hermaphrodite plant with a mall genome size, which can be used for the sex chromosome evolution analysis and species identification of the genus *Asparagus* [5]. Meanwhile, *A. setaceus* has also been proved to be used in Chinese traditional medicine [6]. Considering *A. setaceus* as a closely wild relative species of the most economical vegetable *Asparagus officinalis* in the same genus, it showed strong disease resistance such as rust dot commonly caused by *Puccinia asparagi* [7]. Moreover, we can utilize this important agronomical trait of *A. setaceus* to improve the cultivars of *A. officinalis* by molecular breeding technologies. Therefore, *A. setaceus* shows much importance in our scientific field and horticultural decoration value in ordinary lives for its intrinsic properties.

However, in spite of its great value, there were few genomic resources for *A. setaceus*. Up to now, limited systematic and comprehensive comparative studies of the cp genome was reported in this species, although only one assembled genome of *A. setaceus* has been registered in NCBI (GenBank accession number: NC_047458.1), but this reported genome was released without further sequence analysis. Within the genus, there are five cp genomes released in GenBank (https://www.ncbi.nlm.nih.gov/genome/browse#!/organelles/Asparagus), thus providing valuable genetic information for genomics and phylogeny comparative analysis. In this research, the entire cp genome of *A. setaceus* was de novo sequenced with Illumina and PacBio sequencing technologies. In addition to gene annotation and genome characteristics analysis, we have identified a large number of single-nucleotide polymorphism (SNP) and insertion and deletions (Indels) between our new reported genome and the precious assembly registered in NCBI. Moreover, genomic comparison analysis was carried out with the registered cp genomes of *Asparagus* species, which were useful for the phylogenetic reconstruction, genomic information analysis, and evolutionary research in the genus *Asparagus*.

## 2 Materials and methods

### 2.1 DNA extraction and sequencing

The plant material of *A. setaceus* came from the Department of Biological Technology of Nanchang Normal University (115°27′E, 28°09′N). The genomic DNA of its tender fascicled cladodes was extracted by the improved cetyltrimethyl-ammonium bromide method, using the Qiagen genomic DNA extraction kit (Qiagen, CA, USA) [8]. Based on the manufacturer’s procedure, two libraries with the insert size of 350 bp and 20 kb were constructed individually and then sequenced on an Illumina HiSeq PE150 and a PacBio Sequel sequencing platform at Genepioneer Biotechnologies (Nanjing, China).

### 2.2 Cp genome assembly and annotation

The clean data obtained from the third-generation PacBio sequencing were spliced with Canu software, which included the process of error correction, modification, and assembly [9]. The contigs with coverage >10 were selected for homology search, the cp sequence was determined, and these contigs were screened. Taking the published cp genome sequences of *A. officinalis* (NC_034777.1) and *A. setaceus* (NC_047458.1) in NCBI as a reference genome, the cp data in the whole genome of the sample were isolated by Blastn search and its cp-related reads were assembled with the software Canu. To solve the problem of assembly accuracy in this third-generation sequenced genome, Nextpolish software was used in this study to polish the assembled genome combined with the second-generation Illumina sequencing data [10]. The Illumina reads were assembled with SOAPdenovo2 [11]. The software PGA was used for its annotation [12]. The annotated gene sequence was visualized in Geneious 11.0.3 software [13]. And the annotation was manually corrected to obtain the final result and submitted to GenBank with the serial login number of MT712152.1. Using online OGDRAW1.3.1 software mapped the whole cp genome of *A. setaceus* [14]. In addition, the indels and SNPs detected between the two cp genomes (MT712152.1 and NC_047458.1) were verified by PCR amplification and direct DNA product sequencing (primers used are listed in Table S1). The PCR system was 10 μL, including 1 μL of each forward and reverse primer, 1 μL of genomic DNA (100 ng/μL), 5 μL of 2× EasyTaq^®^ PCR SuperMix (+dye), and 2 μL of deionized water. The PCR procedure was as follows: pre-denaturation at 95°C for 4 min; 35 cycles of 95°C for 30 s, 55°C for 30 s, and 72°C for 30 s; and 72°C for 5 min.

### 2.3 Comparative analysis in cp genome

Based on the Python script prepared by the research group, we counted the cp genome size, LSC, SSC, and IR region size, GC content, total gene number, and gene copy number. Compared with the prior deposited cp genome, the boundary difference of LSC, IR, and SSC regions was determined among five *Asparagus* plants using Mummer 3.0 [15]. Then, the boundaries of LSC, SSC, and IR regions of cp genomes in five *Asparagus* species were visualized by using the SVG module of Perl language, including the expansion and contraction of LSC, IR, and SSC regions, and the gene differences located on the boundaries.

### 2.4 Genome repeats and variation sites

The simple sequence repeat (SSR) sequence with repeat units of 1–6 bases in cp genome was marked out by using the script MISA written in Perl language [16]. The long segment repeats were detected by Reputer in the cp genome [17]. The specific parameter settings containing four types were as follows: forward, reverse, complementary, and palindrome; the shortest repeat unit contained at least 30 bp; and repeat sequence similarity was at least 90%. The cp genome sequences were compared by Mafft software [18]. Based on the comparison results, the mining and visualization of variable outliers were carried out by using Dnasp 6, and the parameters were set by default value [19].

### 2.5 Phylogenetic tree reconstruction

We downloaded the cp genome sequences of 25 species from NCBI (https://www.ncbi. nlm.nih.gov/genome/browse#!/organelles/) in Asparagaceae. Taking *Allium chinense* (NC_043922.1) in the Amaryllidaceae family as the out-group, the total genome sequences in this analysis were compared by Mafft software [18]. The comparison was further optimized by Trimal software to adjust the calculative results [20]. According to the trimmed comparison results, the phylogenetic tree of *A. setaceus* with the maximum likelihood (ML) algorithm was reconstructed using RAxML version 8.0 with the GTRGAMMA model [21]. The bootstrap value was set to 1,000 replicates.

## 3 Results

### 3.1 Cp genome characteristics

**Figure 1:**
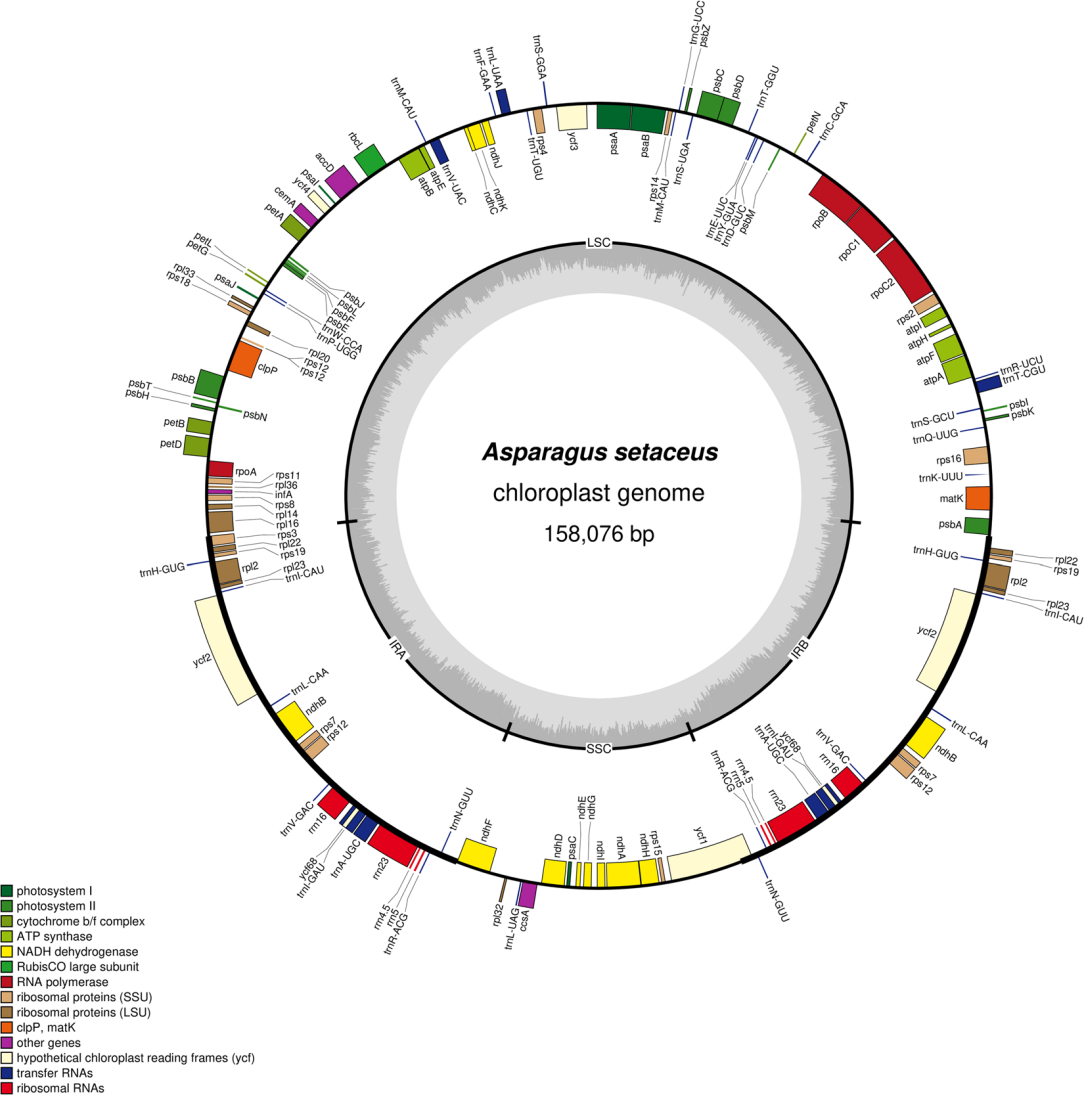
The cp genome map of *A. setaceus*. Note: The genes in the outer ring were arranged clockwise and the genes in the inner ring were arranged counterclockwise in the cp genome. Different colors represented different functions of genes. In the inner cycle, dark gray represented GC content; light gray represented AT content.

The cp genome of *A. setaceus* exhibited a quadripartite structure with a conserved genome arrangement (Figure 1). The cp genome size is 158,076 bp, including a pair of IRs (IRa and IRb, 55,160 bp in total) separated by a LSC region (84,264 bp) and a SSC region (18,652 bp). The GC content of the genome is 37.48%. And the GC content in the IR region (42.6%) was higher than that in LSC (35.45%) and SSC (31.47%), which was in accordance with previous studies [22]. The distribution of four rRNAs in IR region was an important reason for the high GC content in this part [23]. In addition, 135 genes were annotated in *A. setaceus* cp genome, composing of 38 tRNA, 8 rRNA, and 89 protein-coding genes (Table 1). It is reported that introns can regulate the gene transcription rate, which played a vital role in gene structure and function [24]. Statistics showed that 17 genes owned introns in the cp genome of *A. setaceus*. Among them, 10 protein-coding genes and 5 tRNA genes contained 1 intron, and 2 protein-coding genes (*ycf3* and *clpP*) included 2 introns. Furthermore, *rps12* was a *trans*-spliced gene, with 5′ end in the LSC region and 3′ end in the IR region. The length of the introns ranged from 222 to 1,122 bp, among which the intron of *petb* gene was the shortest with the size of 222 bp. And the intron of *ndhA* gene was the longest, which was 1,122 bp in size (Table 2). In addition, the number and type of introns contained in *A. setaceus* were consistent with *A. officinalis*, indicating a highly conserved cp genome of the genus *Asparagus* [25]. And the complete cp genome with gene annotations has been registered under GenBank accession number MT712152.1 for *A. setaceus*.

**Table 1:** Gene annotation and classification in *A. setaceus* cp genome

Category	Gene group	Gene name
Photosynthesis	Subunits of photosystem I	*psaA*, *psaB*, *psaC*, *psaI*, *psaJ*
	Subunits of photosystem II	*psbA*, *psbB*, *psbC*, *psbD*, *psbE*, *psbF*, *psbH*, *psbI*, *psbJ*, *psbK*, *psbL*, *psbM*, *psbN*, *psbT*, *psbZ*
	Subunits of NADH dehydrogenase	ndhA*, ndhB*(2), *ndhC*, *ndhD*, *ndhE*, *ndhF*, *ndhG*, *ndhH*, *ndhI*, *ndhJ*, *ndhK*
	Subunits of cytochrome b/f complex	*petA*, petB*, petD*, *petG*, *petL*, *petN*
	Subunits of ATP synthase	*atpA*, *atpB*, *atpE*, atpF*, *atpH*, *atpI*
	Large subunit of rubisco	*rbcL*
	Subunits photochlorophyllide reductase	—
Self-replication	Proteins of large ribosomal subunit	*rpl14*, rpl16*, rpl2*(2), *rpl20*, *rpl22(2)*, *rpl23(2)*, *rpl32*, *rpl33*, *rpl36*
	Proteins of small ribosomal subunit	*rps11*, rps12**(2), *rps14*, *rps15*, rps16*, *rps18*, *rps19(2)*, *rps2*, *rps3*, *rps4*, *rps7(2)*, *rps8*
	Subunits of RNA polymerase	*rpoA*, *rpoB*, rpoC1*, *rpoC2*
	Ribosomal RNAs	*rrn16(2)*, *rrn23(2)*, *rrn4.5(2)*, *rrn5(2)*
	Transfer RNAs	trnA-UGC*(2), *trnC-GCA*, *trnD-GUC*, *trnE-UUC*, *trnF-GAA*, *trnG-UCC*, *trnH-GUG(2)*, *trnI-CAU(2)*, trnI-GAU*(2), *trnK-UUU*, *trnL-CAA(2)*, trnL-UAA*, *trnL-UAG*, *trnM-CAU(2)*, *trnN-GUU(2)*, *trnP-UGG*, *trnQ-UUG*, *trnR-ACG(2)*, *trnR-UCU*, *trnS-GCU*, *trnS-GGA*, *trnS-UGA*, trnT-CGU*, *trnT-GGU*, *trnT-UGU*, *trnV-GAC(2)*, trnV-UAC*, *trnW-CCA*, *trnY-GUA*
Other genes	Maturase	*matK*
	Protease	clpP**
	Envelope membrane protein	*cemA*
	Acetyl-CoA carboxylase	*accD*
	c-type cytochrome synthesis gene	*ccsA*
	Translation initiation factor	*infA*
	other	—
Genes of unknown function	Conserved hypothetical cp ORF	*ycf1, ycf2(2)*, ycf3**, *ycf4*, *ycf68(2)*

Notes: Gene* (gene with one introns); Gene** (gene with two introns); Gene (2) (gene with two copies).

**Table 2:** The number and length of exons and introns in the cp genome of *A. setaceus.*

Gene	Location	Exon I (bp)	Intron I (bp)	Exon II (bp)	Intron II (bp)	Exon III (bp)
*rps16*	LSC	44	927	136		
*trnT-CGU*	LSC	34	671	45		
*atpF*	LSC	159	841	411		
*rpoC1*	LSC	438	740	1,632		
*ycf3*	LSC	129	750	228	718	153
*trnL-UAA*	LSC	35	527	50		
*trnV-UAC*	LSC	39	585	46		
*rps12*	IRa	120	—	232	544	26
*clpP*	LSC	69	665	291	812	252
*petB*	LSC	6	222	642		
*petD*	LSC	6	728	477		
*rpl16*	LSC	8	960	412		
*rpl2*	IRb	384	664	432		
*ndhB*	IRb	777	699	756		
*rps12*	IRb	232	—	26	544	114
*trnI-GAU*	IRb	42	936	35		
*trnA-UGC*	IRb	38	815	35		
*ndhA*	SSC	558	1,122	540		
*trnA-UGC*	IRa	38	815	35		
*trnI-GAU*	IRa	42	936	35		
*ndhB*	IRa	777	699	756		
*rpl2*	IRa	384	664	432		

### 3.2 Genome variation

Contrast our new assembled genome with the prior registered genome in GenBank (NC_047458.1), we detected a number of variations including 7 SNPs (6 transversions and 1 transitions) and 16 indels (from 1 to 3 bp) between the two genomes. To further confirm the existence of these mutation sites, 23 pairs of primers were further designed to verify the existence of these mutation sites (Table S1). Among the variations, 2 SNPs and 13 indels were found in the LSC regions, 2 SNPs and 1 indels were marked out within the SSC region, 1 SNP and 1 indel were detected in the IRa region, and 2 SNPs and 1 indel were checked in the IRb region (Table 3). And nearly all the variations were positioned in noncoding regions consisting of intergenic spacer (IGS) and intron sequences, except two variations that were found in the *rpoC1* and *rps15* genes. From the above results, we can conclude that the variation in LSC region was the largest (65.22%), the variation in IR region was the second (21.74%), and the variation in SSC region was the smallest (13.04%) in *A. setaceus* cp genome. It was also found that the variation in noncoding region sequence (91.3%) was much greater than that in the coding region (8.7%).

**Table 3:** SNP and Indel difference between the new registered MT712152.1 and the previous NC_047458.1 of *A. setaceus* cp genome in NCBI

No.	Type	Region	Position^a^	Location	MT712152.1	NC_047458.1	Gene
1	Indel	LSC	3,363	*trnK-UUU*-intron (N^b^)	C	CTT	
2	Indel	LSC	4,934	*trnK-UUU*-*rps16* (N)	C	CC	
3	Indel	LSC	8,509	*trnS-GCU* (N)	T	TAA	
4	Indel	LSC	8,654	*trnS-GCU* (N)	G	GA	
5	SNP	LSC	22,410	*rpoC1*(C^c^) (N)	T	G	*rpoC1*
6	Indel	LSC	28,007–28,008	*trnC-GCA* (N)	TA	T	
7	SNP	LSC	42,876	*psaA* (N)	G	C	
8	Indel	LSC	60,326–60,326	*ycf4*-intron (N)	C	CT	
9	Indel	LSC	60,848	*ycf4*-intron (N)	T	TAA	
10	Indel	LSC	70,764	*rpl20-rps12* (N)	C	CA	
11	Indel	LSC	71,986	*rps12*-intron (N)	G	GA	
12	Indel	LSC	73,101–73,102	*clpP*-intron (N)	GC	G	
13	Indel	LSC	77,858	petB-intron(N)	G	GA	
14	Indel	LSC	78,673–78,674	*petD*-intron (N)	CG	C	
15	Indel	LSC	82,133	*rpl14-rpl16* (N)	T	TAA	
16	SNP	IRA	107,283	t*rnA-UGC-rrn23* (N)	G	A	
17	Indel	IRA	110,166	*trnN-GUU-*intron (N)	C	CTT	
18	SNP	SSC	114,478	*ycf1*-intron (N)	C	T	
19	Indel	SSC	114,489	*ycf1*-intron (N)	G	GA	
20	SNP	SSC	126,686	*rps15*-intron (N)	T	G	*rps15*
21	Indel	IRB	132,569	*rps15*-intron (N)	T	TAA	
22	SNP	IRB	133,089	*trnA-UGC-trnV-GAC* (N)	A	C	
23	SNP	IRB	141,598	*trnV-GAC*-intron (N)	A	C	

^a^Nucleotide position is referenced to our new assembly. ^b^N, noncoding sequences including IGS region and intron. ^c^Coding sequences.

### 3.3 Codon usage bias and RNA editing sties predication

**Figure 2:**
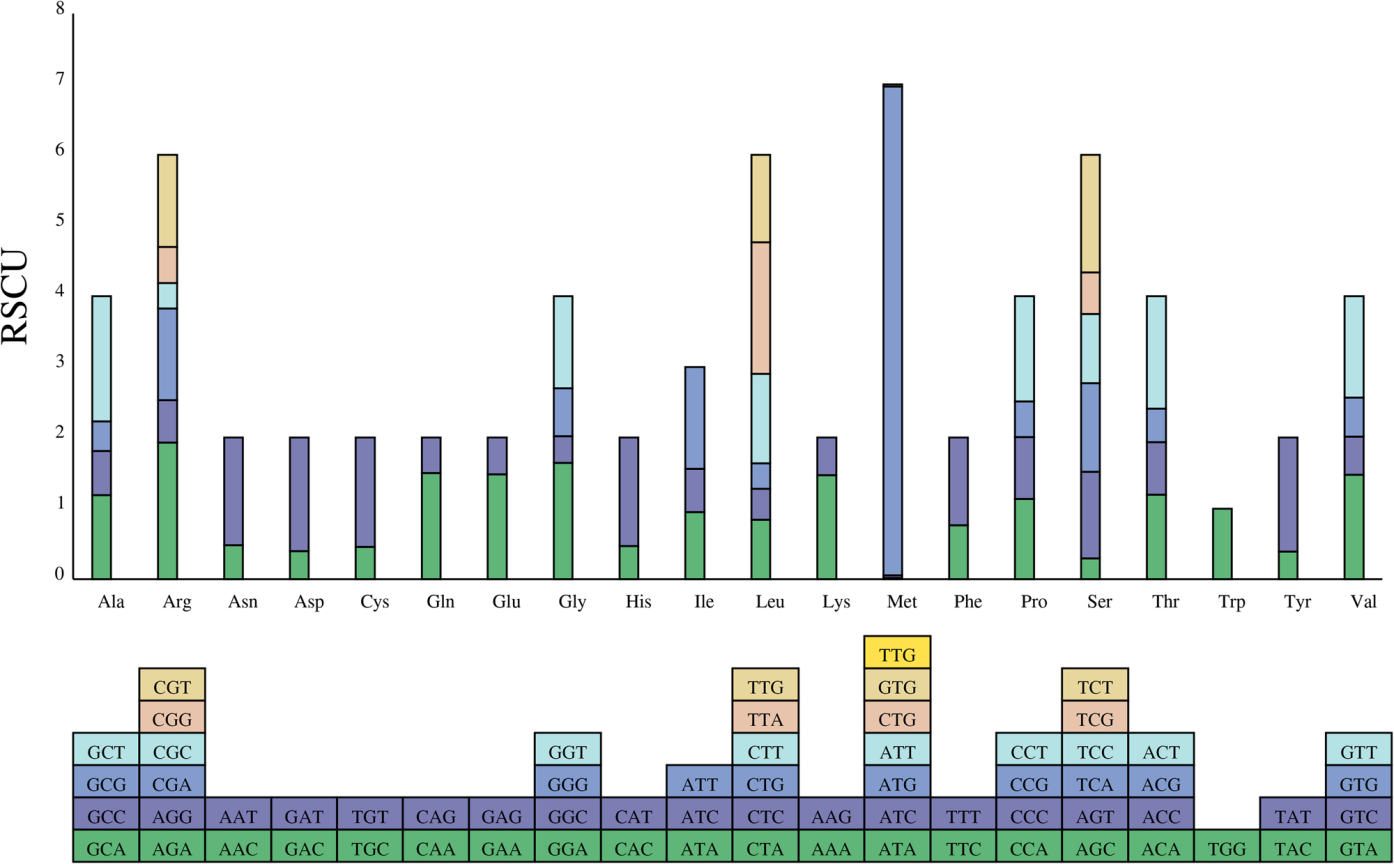
The RSCU histogram in the cp genome of *A. setaceus.* Note: the *Y*-axis represents the value of RSCU, the *X*-axis represents the type of amino acids, and the following block represents the codon encoding each amino acid.

The relative synonymous codon usage (RSCU) was calculated in the cp genome of *A. setaceus* with Codon W1.4.2 (https://sourceforge.net/projects/codonw/files/OldFiles/CodonWSourceCode_1_4_2.tar.gz/download). Using the method proposed by Wright [26], 52 (coding sequence, CDs) sequences meeting the requirements were finally selected from 89 CDs annotated in the cp genome of *A. setaceus* for further analysis. According to the analysis results, leucine (2757, 10.31%) was the largest number amino acid among the proteins encoded by the cp genes, followed by Ile (2287, 8.55%) and Ser (2094, 7.83%). Cysteine (316, 1.18%) was the least abundant amino acid among the proteins encoded by the cp genes in *A. setaceus* genome (Figure 2). Leucine and isoleucine were the most commonly observed amino acids in the cp genome proteins. And usage of the codon UGG (tryptophan) had no bias (RSCU = 1). All preferred relative synonymous codons (RSCU > 1) ended with A or U.

**Figure 3:**
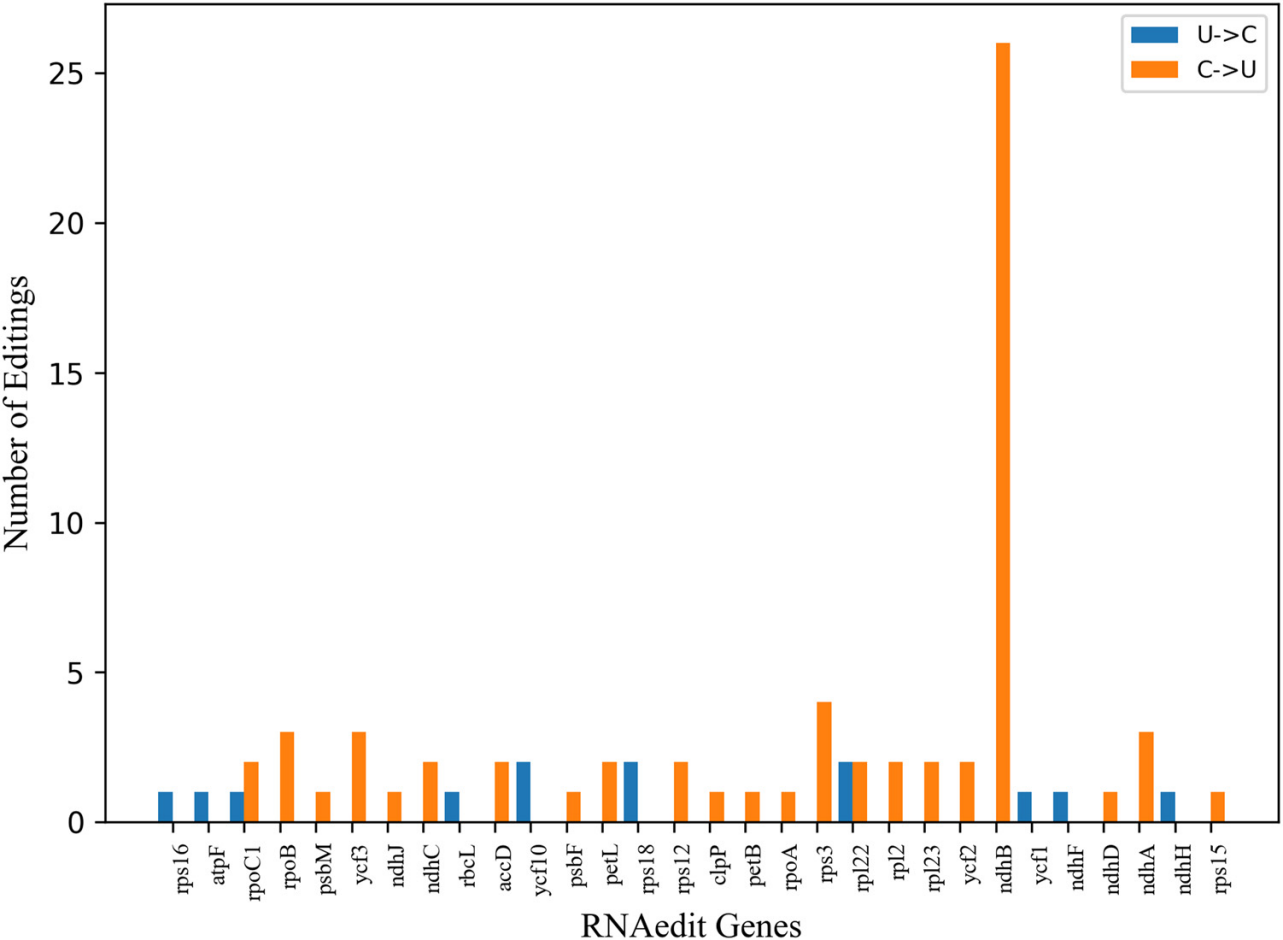
The value of RNA edit genes in the cp genome of *A. setaceus*. Note: Abscissa refers to different gene types; the ordinate refers to the number of RNA editing sites.

To gain insights into the RNA-editing sites in *A. setaceus*, 78 RNA editing sites of 29 cp genes were calculated with the PREP suite [27]. The result showed the number of editing sites was from 1 to 26, of which *ndhB* contained the largest number of editing sites. And most genes had one site, accounting for 46.67%. There were two types of editing sites, U–C and C–U, which were 16.67% and 83.33% respectively. Among the variation types of amino acids, the maximum number of serine (S)–leucine (L) was 37, accounting for 30.83% (Figure 3). It was seen that the amino acid conversion from S to L was the most frequent type. As previously reported, the conversion from S to L became more frequent along with the number increasing of amino acids [28]. This finding indicated that the amino acid conversion was essential in RNA editing during the evolutionary process.

### 3.4 Repeat sequence analysis

Long repeats greater than or equal to 30 bp were considered playing a key role in genome rearrangement [29,30]. In *A. setaceus*, there were 36 repeats including 13 forward repeats, 2 reverse repeats, and 21 palindrome repeats, without complementary repeats (Table 4). The length distribution was mainly 30–56 bp in the repeat sequence; the longest repeat was 27,580 bp, positioned in the IR region; and the shortest repeat was 30 bp, containing 12 sites. According to the quadripartite structure in the cp genome, IR regions had the most repeats (16, 44.45%), followed by LSC region (12, 33.33%), SSC region (6, 16.67%), and the overhanging junction region (2, 5.55%). Based on the classification of gene structure, a majority of the repeat sites were located in IGS regions, in which the *ycf2*-IGS area contained the most numbers of repeat sites (4, 11.11%). And only a few types of genes (*ycf1*, *ycf2*, *ycf3*, *psaB*; *psaA*, *trnS-GCU*, *trnS-GGA*, *atpF*, *trnS-UGA*, *trnS-GGA*, *trnT-CGU*, *trnG-UCC*) possessed repeat elements, and *ycf2* had the highest number of repeat sites (11, 30.56%).

**Table 4:** Repeat sequences in the *A. setaceus* cp genome

ID	Repeat I start	Repeat II start	Type	Size (bp)	Distance	*E*-value	Gene	Region
1	84,265	130,497	P	27,580	0	0.00 × 10^00^	—	IR
2	29,819	29,819	P	56	0	1.35 × 10^−24^	IGS	LSC; LSC
3	90,646	90,667	F	49	−1	3.26 × 10^−18^	*ycf2*; *ycf2*	IRb; IRb
4	90,646	151,626	P	49	−1	3.26 × 10^−18^	*ycf2; ycf2*	IRb; IRa
5	90,667	151,647	P	49	−1	3.26 × 10^−18^	*ycf2; ycf2*	IRb; IRa
6	151,626	151,647	F	49	−1	3.26 × 10^−18^	*ycf2; ycf2*	IRa; IRa
7	39,149	41,373	F	47	−3	1.55 × 10^−13^	*psaB; psaA*	LSC; LSC
8	44,084	100,421	F	39	−3	5.74 × 10^−9^	*ycf3*; IGS	LSC; IRb
9	44,084	141,882	P	39	−3	5.74 × 10^−9^	*ycf3*; IGS	LSC; IRa
10	125,629	125,629	P	39	−3	5.74 × 10^−9^	IGS	SSC; SSC
11	126,988	126,988	P	39	−3	5.74 × 10^−9^	*ycf1; ycf1*	SSC; SSC
12	129,357	129,372	F	39	−3	5.74 × 10^−9^	*ycf1; ycf1*	SSC; SSC
13	8786	8786	P	38	0	9.30 × 10^−14^	IGS	LSC; LSC
14	32,202	32,223	P	37	−3	7.81 × 10^−8^	IGS	LSC; LSC
15	46,984	46,987	P	37	−3	7.81 × 10^−8^	IGS	LSC; LSC
16	127,224	127,224	R	34	−2	1.20 × 10^−7^	*ycf1; ycf1*	SSC; SSC
17	8222	45,499	P	33	−2	4.53 × 10^−7^	*trnS-GCU; trnS-GGA*	LSC; LSC
18	12,664	13,097	P	33	−3	1.40 × 10^−5^	*atpF; atpF*	LSC; LSC
19	115,799	115,799	R	33	−2	4.53 × 10^−7^	IGS	SSC; SSC
20	69,877	69,893	F	32	−2	1.70 × 10^−6^	IGS	LSC; LSC
21	8220	35,994	F	32	−3	5.10 × 10^−5^	*trnS-GCU; trnS-UGA*	LSC; LSC
22	35,997	45,499	P	32	−3	5.10 × 10^−5^	*trnS-UGA; trnS-GGA*	LSC; LSC
23	9970	36,975	F	31	−3	1.85 × 10^−4^	*trnT-CGU; trnG-UCC*	LSC; LSC
24	114,879	114,879	P	31	−3	1.85 × 10^−4^	IGS	SSC; SSC
25	46,996	46,996	P	30	−2	2.39 × 10^−5^	IGS	LSC; LSC
26	90,668	90,689	F	30	−2	2.39 × 10^−5^	*ycf2; ycf2*	IRb; IRb
27	90,668	151,623	P	30	−2	2.39 × 10^−5^	*ycf2; ycf2*	IRb; IRa
28	90,689	151,644	P	30	−2	2.39 × 10^−5^	*ycf2; ycf2*	IRb; IRa
29	88,000	88,023	F	30	−3	6.68 × 10^−4^	IGS; *ycf2*	IRb; IRb
30	88,000	154,289	P	30	−3	6.68 × 10^−4^	IGS; *ycf2*	IRb; IRa
31	88,023	154,312	P	30	−3	6.68 × 10^−4^	*ycf2*; IGS	IRb; IRa
32	90,644	90,686	F	30	−3	6.68 × 10^−4^	*ycf2; ycf2*	IRb; IRb
33	90,644	151,626	P	30	−3	6.68 × 10^−4^	*ycf2; ycf2*	IRb; IRa
34	90,686	151,668	P	30	−3	6.68 × 10^−4^	*ycf2; ycf2*	IRb; IRa
35	151,623	151,665	F	30	−3	6.68 × 10^−4^	*ycf2; ycf2*	IRa; IRa
36	154,289	154,312	F	30	−3	6.68 × 10^−4^	*ycf2*; IGS	IRa; IRa

**Figure 4:**
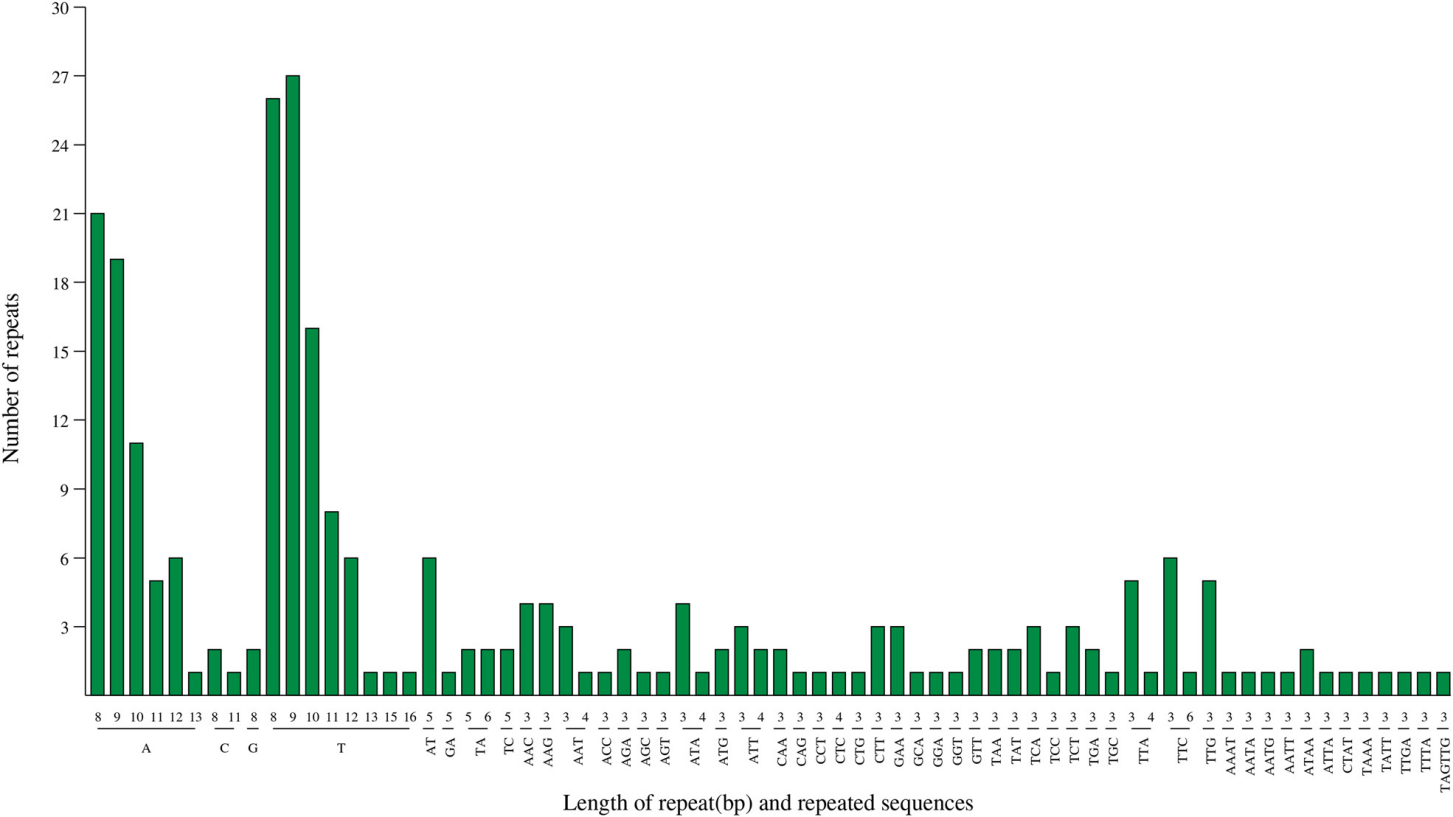
The type and number of SSR motif in the *A. setaceus* cp genome.

Tandem repeat sequences were known as SSRs or microsatellites, usually consisting of 1–6 nucleotide repeat units. The majority of SSRs were mono- and tri-nucleotide repeats in *A. setaceus* cp genome, which had the number of 155 and 79 times, respectively. The mononucleotide repeats were almost A/T repeats (96.15%), and 76.92% of the dinucleotide repeats were AT/TA repeats. SSRs in cp genome of *A. setaceus* also preferred to use A/T bases, which was in line with previous studies on *A. officinalis*, that is, SSR markers in plant cps were rich in A/T repeats [25,31]. And 13 di-nucleotide, 12 tetra-nucleotide, and only one hexa-nucleotide SSRs were detected (Figure 4). The length of repeated sequences was found to range from 8 to 16 bp, similar with the lengths reported in other angiosperm plants [32]. Therefore, the high variation in SSRs in the *A. setaceus* cp genome is of great value for the development of molecular marker studies.

### 3.5 Non-synonymous/synonymous substitution value analysis

**Figure 5:**
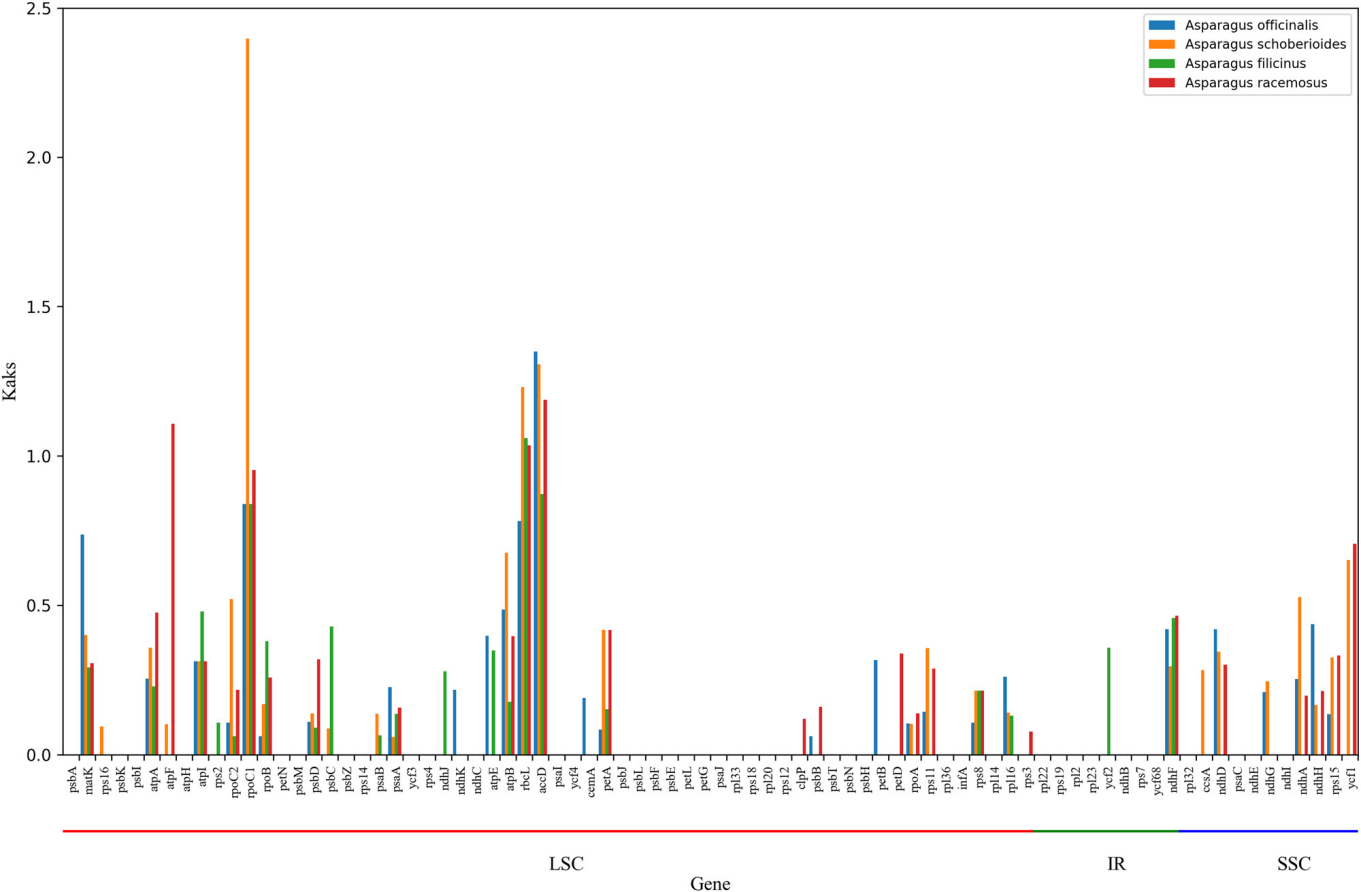
The *K*_a_/*K*_s_ ratio of 80 protein-coding genes of the *A. setaceus* genome and four closely related species in the genus *Asparagus.*

To further study the selection pressure on cp genes of *A. setaceus* and other *Asparagus* species in the process of evolution, the *K*_a_/*K*_s_ values of protein-coding genes in *A. setaceus* vs *A. officinalis*, *A. setaceus* vs *Asparagus schoberioides*, *A. setaceus* vs *Asparagus filicinus*, and *A. setaceus* vs *Asparagus racemasus* were calculated by Dnasp software individually [19] (Figure 5). In total, 80 protein-coding genes were analyzed. The *K*_a_/*K*_s_ average value was 0.1962, 0.2413, 0.1836, and 0.2547, respectively, and most of the genes had *K*_a_/*K*_s_ < 1, which showed that the cp genes of the *Asparagus* species had been strongly purified and selected in the long-term evolution process.

### 3.6 IRScope analysis

**Figure 6:**
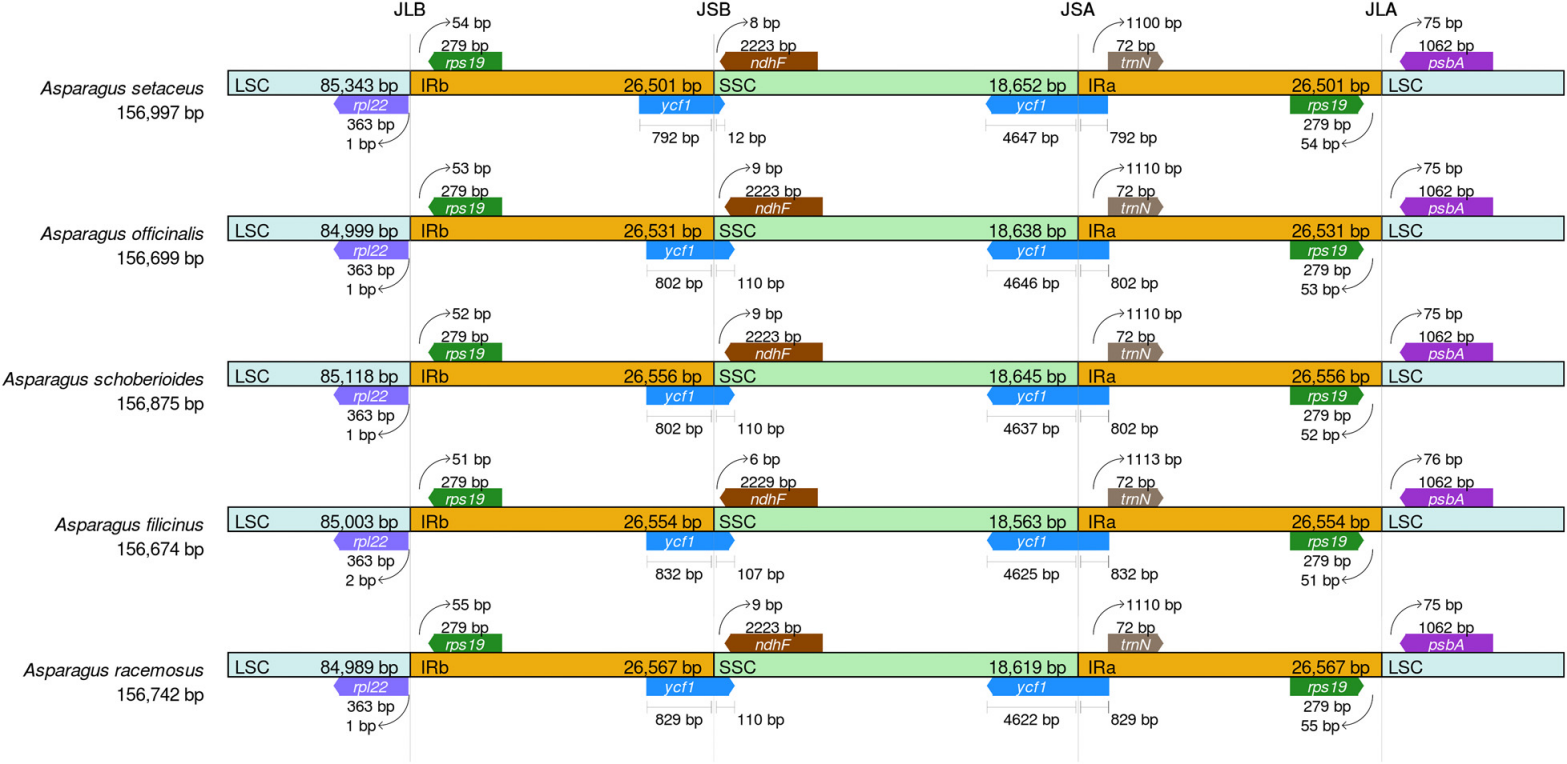
Comparative analysis of LSC, SSC, and IR regional boundaries of five *Asparagus* species.

The study showed that there were four boundaries in the cp genome of the *Asparagus* species, namely containing LSC region-inverted region b (LSC-IRb), inverted region b-SSC region (IRb-SSC), SSC region-inverted region a (SSC-IRa), and inverted region a-LSC region (IRa-LSC). The cp genome structure of the five selected *Asparagus* plants was relatively conservative (Figure 6). It was found that the boundaries between these species were consistent, and the difference was the length of genes from the boundary.

### 3.7 Genome comparative analysis

**Figure 7:**
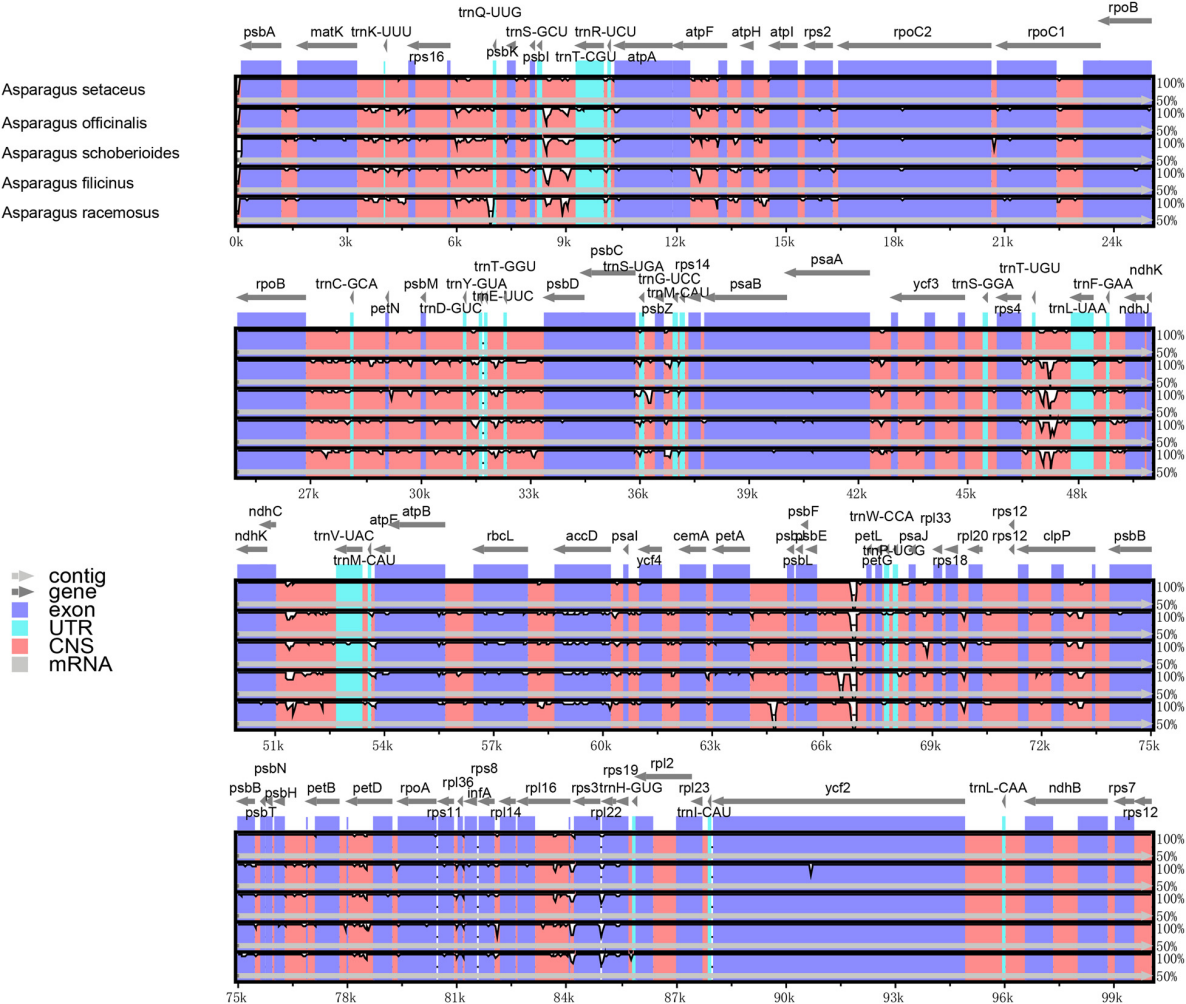
Sequence alignment of five cp genome in the genus Asparagus by mVISTA, with the annotation of *A. setaceus as* the reference.

The five known cp genome sequences in the genus *Asparagus* were compared. The result indicated that species with the largest genome was *A. officinalis* and that with the smallest was *A. setacus*. The gene order and content in the cp genome were used to analyze its difference with the online program mVISTA (https://genome.lbl.gov/vista/vista/bout.html). The gene order and contents of the *Asparagus* plants were found to be similar with those of other members in the genus *Asparagus* (Figure 7). It can be seen that all *Asparagus* species had conserved cp genomes, their coding regions were more conserved than their noncoding regions, and their IR regions were more conserved than their LSC and SSC regions.

### 3.8 Phylogenetic relationship reconstruction

**Figure 8:**
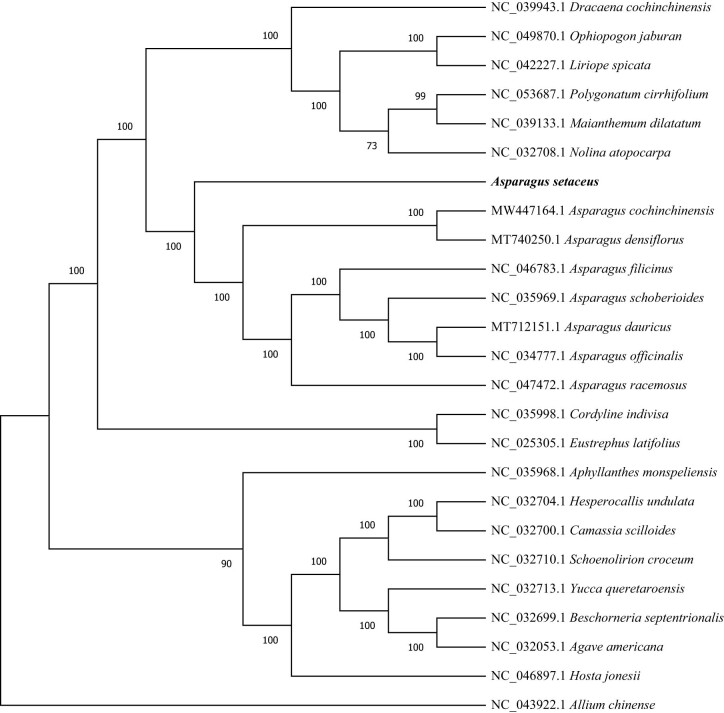
Phylogenetic tree was built based on the entire cp genomes from 25 species using RAxML. with GTRGAMMA model. And *A. chinense* was set as the out-group. Bootstrap values were displayed at the nodes.

The cp genome contains abundant information, and its structure, size, and gene composition are relatively constant, which has been widely utilized in phylogenetic analysis and species identification [33]. The cp genome can be used to resolve the deeper branches within species. To straighten out the phylogenetic positions of *A. setaceus* within the genus *Asparagus*, the ML method of phylogenetic analysis was performed based on the complete cp genome dataset from 24 plant taxa, with *A. chinense* used as the out-group. The ML tree had similar phylogenetic topologies, and most nodal support values were high. The higher was the branch’s credibility, the more consistent was the guiding value of the evolutionary analysis for the relationship [1]. Furthermore, the phylogenetic tree suggested that *A. setaceus* formed a single group, *Asparagus cochinchinensis* and *Asparagus densiflorus* were grouped into another group, and they were sister groups with a support rate of 100% (Figure 7). This was similar to Norup’s research result [34]. It was speculated that *A. setaceus* belonged to the subgenus *Asparagopsis* derived from the African origin, which had a certain genetic distance from other sub-genus *Asparagus* group in Asia (Figure 8).

## 4 Discussion

There are generally two traditional methods for obtaining plant cp genome. One is to isolate cp organelles from plant tissues, then extract cp DNA, and obtain plant cp genome with the first- or second-generation sequencing technology. But it is difficult to isolate whole cps and obtain high-quality cp DNA. The other method is to extract the whole plant genome DNA and then use the conserved region of cp genome to design primers with the first-generation sequencing method and finally splice the plant cp genome. The disadvantage of this method is that it is difficult to obtain complete cp genome sequence [32]. Along with the development of the new-generation sequencing technology, especially the second- and third-generation sequencing technology, and the extensive use of a large number of Bioinformatics software, the whole genome DNA of plants can be extracted for high-throughput sequencing, and the cp reads of the samples are extracted and assembled to obtain the cp genome of plants. This method does not require the separation of cp DNA, reduces the labor intensity, and improves the success rate of the experiment [35]. The Illumina HiSeq second-generation and PacBio Sequel third-generation sequencing platforms have high flux, and this method can effectively obtain the cp genome under the premise of containing cp sequences from related species [36]. Therefore, Illumina HiSeq sequencing platform was used to re-sequence the whole genome of *A. setaceus* and the cp genome of *A. setaceus* was assembled with related species by the software Canu and SOAPdenovo2 in this study, which provided a successful example for cp genome sequencing and assembly annotation of other species.

In the genus *Asparagus*, it belongs to a group of commonly used Chinese medicinal materials. Many medicinal plants are under great pressure of artificial selection in the long-term selection process, resulting in the similarity of many plants in this group, which is difficult to distinguish and identify [5,7]. Therefore, the study of cp genome is of great value to the genetic research of this genus. To detect the differences between the cp genomes of the genus *Asparagus*, four published species (*A. filicinus*, *A. schoberioides*, *A. officinalis*, and *Asparagus racemosus*) were downloaded from GenBank for comparison. The results showed that there was little difference in the length of cp genome between *A. setaceus* and its related species, with the length between 156,674 and 157,119 bp, and the type and number of genes were roughly the same, which proved that the cp genome was highly conserved. The length difference of cp genome in *Asparagus* plants mainly occurred in LSC region, which may be caused by the insertion and deletion of gene spacer, which was in line with the cp genome of most angiosperms [37].

On the basis of obtaining the structure and composition in *A. setaceus* cp genome, this study analyzed its codon preference, repeat sequence, SSR characteristics, boundary differences, and polymorphism sites, which provided a data basis for the study of cp genome in this genus. Phylogenetic analysis showed that *A. setaceus* was closely related to *A. cochinchinensis* and *A. densiflorus*. Due to the close genetic relationship of *Asparagus* plants, inter-specific hybridization within the genus was easy, and the intermediate type and transitional type were quite common, so the systematic classification was difficult [38]. The use of cp genome can provide a reference for the classification of plants in the genus, but the number of published cp genomes in the genus *Asparagus* is still very limited (https://www.ncbi.nlm.nih.gov/genome/browse#!/Organelles/Asparagus); the relevant research only stays in the comparative analysis of different species cp genomes. Therefore, it is necessary to obtain more cp genomes of this genus to better solve the phylogenetic problem of the genus *Asparagus* in Asparagaceae.

## 5 Conclusion

Through the methods of second-generation and third-generation sequencing platform, combined with the homology sequence alignment of related species and the use of cp splicing software, the whole cp genome sequence can be obtained. This program establishes a reference for the report of cp genome in other species. In this research, a typical quadripartite structure was exhibited in *A. setaceus* cp genome with 158,076 bp, including 89 protein-coding, 38 tRNA, and 8 rRNA genes. Contrast with the previous *A. setaceus* cp genome in NCBI, we had detected 7 SNPs and 16 Indels, which were mostly distributed in noncoding areas. In addition, 260 SSRs and 36 repeat sequences marked out in the cp genome could be utilized for species identification. Furthermore, A/T ending bias was detected and C-to-U transitions were found for the identified RNA editing sites in this cp genome. It was also seen that the cp genome had similarity with the sequenced species in genome size, gene composition, and genetic organization in the genus *Asparagus*. By the phylogenetic reconstruction of the whole cp genome, it was shown that *A. setaceus* was closely related with *A. cochinchinensis* in the genus. Therefore, the reported cp genome provided information for sequence variation, genomic comparison, and phylogenetic relationship studies in Asparagaceae.

## Supplementary Material

Supplementary Table
